# 1-Phenyl-1-[(1-phenyl­ethyl)sulfonyl­methyl­sulfon­yl]ethane

**DOI:** 10.1107/S1600536809049423

**Published:** 2009-11-25

**Authors:** Bing-zhu Zhang, Xiang-min Chen, Huai-ping Kang, Feng-xia Sun, Yan-ji Wang, Jing-tao Liu, De-bin Wang

**Affiliations:** a College of Chemical Engineering, Hebei University of Technology, Tianjin 300130, People’s Republic of China; b College of Chemical and Pharmaceutical Engineering, Hebei University of Science and Technology, Shijiazhuang 050018, People’s Republic of China; c Graduate School, Hebei University of Science and Technology, Shijiazhuang 050018, People’s Republic of China

## Abstract

There are two mol­ecules in the asymmetric unit of the title compound, C_17_H_20_O_4_S_2_. There are slight differences in the twist of the two rings relative to the S–C–S chain [dihedral angles of 48.41 (18) and 87.58 (16)° in the first mol­ecule and 45.98 (18) and 87.02 (18)° in the second] and the difference in the C—S—C—S torsion angles [176.68 (17) and −77.6 (2)° for the two independent mol­ecules].

## Related literature

The title compound is a by-product from the synthesis of 1-phenyl­ethanesulfonic acid. 1-Phenyl-ethanesulfonic acid is a favorable resolving agent for the diastereomeric resolution of dl-*p*-hydroxy­phenyl­glycine, see: Yoshioka, *et al.* (1987 ▶). d-*p*-hydroxy­phenyl­glycine is useful as a side chain in semi-synthetic penicillins or cephalosporins, see: Crast (1970 ▶).
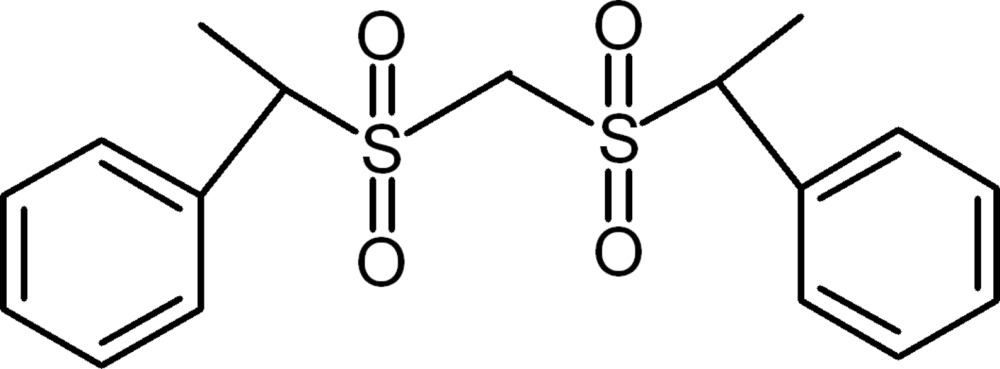


## Experimental

### 

#### Crystal data

C_17_H_20_O_4_S_2_
*M* *_r_* = 352.45
Orthorhombic, 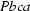

*a* = 16.662 (3) Å
*b* = 19.270 (4) Å
*c* = 22.094 (4) Å
*V* = 7094 (2) Å^3^
*Z* = 16
Mo *K*α radiation
μ = 0.32 mm^−1^
*T* = 293 K
0.20 × 0.18 × 0.16 mm

#### Data collection

Rigaku Saturn CCD area-detector diffractometer
Absorption correction: multi-scan (*CrystalClear*; Rigaku, 2005 ▶) *T*_min_ = 0.940, *T*_max_ = 0.951
45855 measured reflections
6253 independent reflections
5060 reflections with *I* > 2σ(*I*)
*R*_int_ = 0.052

#### Refinement

*R*[*F* ^2^ > 2σ(*F* ^2^)] = 0.058
*wR*(*F* ^2^) = 0.155
*S* = 1.11
6253 reflections
420 parameters
H-atom parameters constrained
Δρ_max_ = 0.26 e Å^−3^
Δρ_min_ = −0.31 e Å^−3^

### 

Data collection: *CrystalClear* (Rigaku, 2005 ▶); cell refinement: *CrystalClear* data reduction: *CrystalClear*; program(s) used to solve structure: *SHELXS97* (Sheldrick, 2008 ▶); program(s) used to refine structure: *SHELXL97* (Sheldrick, 2008 ▶); molecular graphics: *SHELXTL* (Sheldrick, 2008 ▶); software used to prepare material for publication: *SHELXTL*.

## Supplementary Material

Crystal structure: contains datablocks global, I. DOI: 10.1107/S1600536809049423/fl2264sup1.cif

Structure factors: contains datablocks I. DOI: 10.1107/S1600536809049423/fl2264Isup2.hkl

Additional supplementary materials:  crystallographic information; 3D view; checkCIF report

## Figures and Tables

**Fig. 1.**
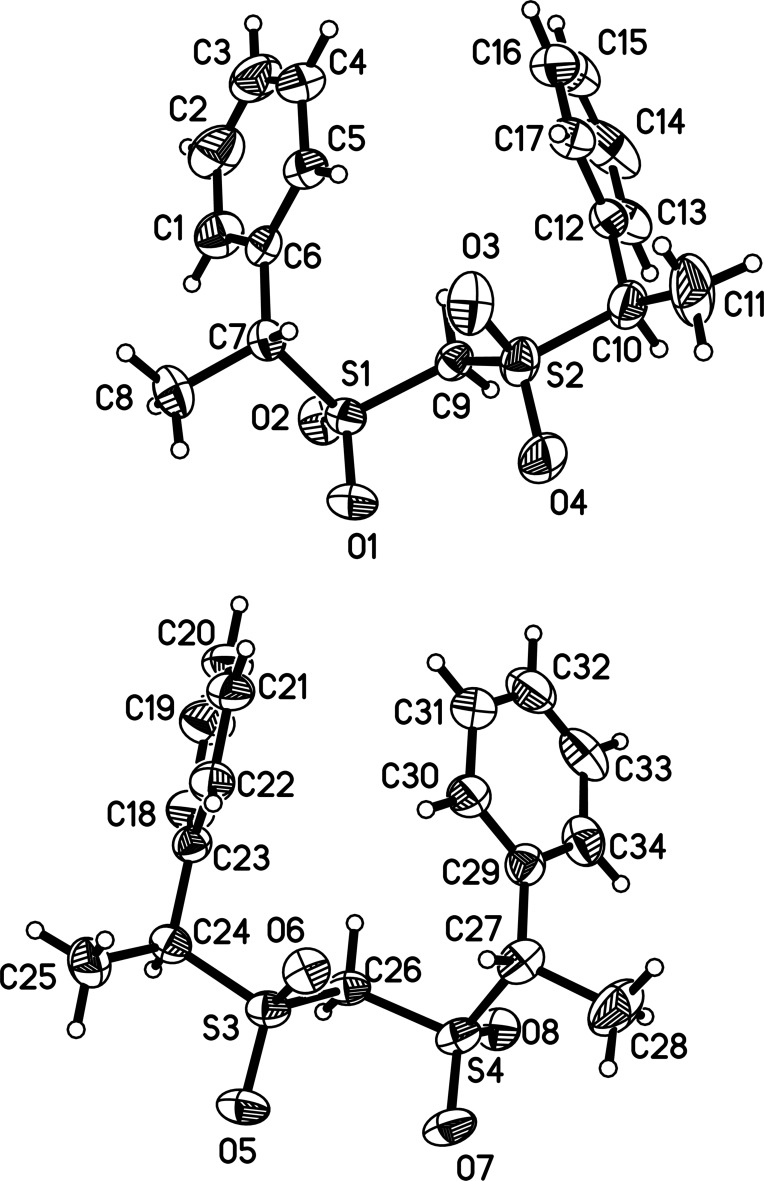
A view of the title compound (I). Displacement ellipsoids are drawn at the 30% probability level and H atoms are shown as small spheres of arbitrary radii.

## supplementary crystallographic information

### Comment

It is well known that *D*-*p*-hydroxyphenylglycine is useful as a side chain of semisynthetic penicillins or cephalosporins (Crast, 1970). 1-Phenyl-ethanesulfonic acid is a favorable resolving agent for the diastereomeric resolution of DL-*p*-hydroxyphenylglycine ( Yoshioka, *et al.*,1987). The title compound (Fig. 1) is a byproduct from the synthesis of 1-phenyl- ethanesulfonic acid. The compound crystallized with two molecules per asymmetric unit. The formula is symmetric about the central carbon but there is no physical symmetry relating the two halves of the molecule. The dihedral angle between the two aromatic rings is essentially the same in each molecule. The crystallographic symmetry was lost due to the small differences in the twist of the two rings related to the CSCSC chain and also the difference in the two CSCS torsion angles in the chain itself (176.68 (17) and -77.6 (2) for the two independent molecules). The dihedral angle between the C10-C12-C13-C14-C15-C16-C17 plane and the plane formed by atoms S2-C9-S1 is 87.58 (16), while the dihedral angle between the C1-C2-C3-C4-C5-C6-C7 plane and the plane formed by atoms S2-C9-S1 is 48.41 (18). The dihedral angle between C27-C29-C30-C31-C32-C33-C34 plane and the plane formed by atoms S4-C26-S3 is 45.98 (18), while the dihedral angle between C18-C19-C20-C21-C22-C23-C24 plane and the plane formed by atoms S4-C26-S3 is 87.02 (18). C10 and C7 are displaced from the plane formed by atoms S2-C9-S1 by 0.102 (1) Å and 1.668 (1) Å respectively while in the second molecule, C27 and C24 are displaced from the plane formed by atoms S4-C26-S3 by 1.695 (1) Å and 0.123 (1) Å respectively..

### Experimental

The title compound was found by chance during the synthesis of 1-phenyl- ethanesulfonic acid. In order to clarify the structure, we prepared the compound as following. 1-Phenyl-ethanethiol (10 g), paraformaldehyde(1.5 g) and water(4 mL) were placed in a 50 mL three-necked flask.The mixture was cooled to 283 K.To this suspension, hydrochloric acid (30%,10 g)was added at 283 K. After the addition was completed, the solution was stirred until all the thiol had reacted (according to TLC, 7 h). The solution was then extracted with petroleum ether(80 mL). The combined organic layers was dried over anhydrous magnesium sulfate, and concentrated *in vacuo* to yield a brown liquid (8.9 g). Hydrogen peroxide(30%, 28.0 g) was added to the solution of the brown liquid (8.9 g) and acetic acid(11.1 g) at 283 K. The solution was stirred at 283 K for 4 h to obtain the title compound. Ethyl acetate was used to extract and light yellow block crystals were obtained by slow evaporation of the solution.

### Refinement

H atoms on C atoms were placed in calculated positions and constrained to ride on their parent atoms, with C—H = 0.93–0.98 A ° and *U*_iso_(H) = 1.2Ueq(C) or 1.5Ueq(methyl C).

### Crystal data

**Table:**

C_17_H_20_O_4_S_2_	*D*_x_ = 1.320 Mg m^−^^3^
*M**_r_* = 352.45	Melting point = 443.0–442.0 K
Orthorhombic, *P**b**c**a*	Mo *K*α radiation, λ = 0.71073 Å
*a* = 16.662 (3) Å	Cell parameters from 12971 reflections
*b* = 19.270 (4) Å	θ = 2.1–27.1°
*c* = 22.094 (4) Å	µ = 0.32 mm^−^^1^
*V* = 7094 (2) Å^3^	*T* = 293 K
*Z* = 16	Block, light yellow
*F*(000) = 2976	0.20 × 0.18 × 0.16 mm

### Data collection

**Table:**

Rigaku Saturn CCD area-detector diffractometer	6253 independent reflections
Radiation source: rotating anode	5060 reflections with *I* > 2σ(*I*)
confocal	*R*_int_ = 0.052
Detector resolution: 7.31 pixels mm^-1^	θ_max_ = 25.0°, θ_min_ = 2.1°
ω and φ scans	*h* = −19→18
Absorption correction: multi-scan (*CrystalClear*; Rigaku, 2005)	*k* = −22→22
*T*_min_ = 0.940, *T*_max_ = 0.951	*l* = −26→26
45855 measured reflections	

### Refinement

**Table:**

Refinement on *F*^2^	Secondary atom site location: difference Fourier map
Least-squares matrix: full	Hydrogen site location: inferred from neighbouring sites
*R*[*F*^2^ > 2σ(*F*^2^)] = 0.058	H-atom parameters constrained
*wR*(*F*^2^) = 0.155	*w* = 1/[σ^2^(*F*_o_^2^) + (0.0766*P*)^2^ + 2.0257*P*] where *P* = (*F*_o_^2^ + 2*F*_c_^2^)/3
*S* = 1.11	(Δ/σ)_max_ = 0.002
6253 reflections	Δρ_max_ = 0.26 e Å^−^^3^
420 parameters	Δρ_min_ = −0.31 e Å^−^^3^
0 restraints	Extinction correction: *SHELXL97* (Sheldrick, 2008), Fc^*^=kFc[1+0.001xFc^2^λ^3^/sin(2θ)]^-1/4^
Primary atom site location: structure-invariant direct methods	Extinction coefficient: 0.0035 (4)

### Special details

**Table:**

Geometry. All e.s.d.'s (except the e.s.d. in the dihedral angle between two l.s. planes) are estimated using the full covariance matrix. The cell e.s.d.'s are taken into account individually in the estimation of e.s.d.'s in distances, angles and torsion angles; correlations between e.s.d.'s in cell parameters are only used when they are defined by crystal symmetry. An approximate (isotropic) treatment of cell e.s.d.'s is used for estimating e.s.d.'s involving l.s. planes.
Refinement. Refinement of *F*^2^ against ALL reflections. The weighted *R*-factor *wR* and goodness of fit *S* are based on *F*^2^, conventional *R*-factors *R* are based on *F*, with *F* set to zero for negative *F*^2^. The threshold expression of *F*^2^ > σ(*F*^2^) is used only for calculating *R*-factors(gt) *etc*. and is not relevant to the choice of reflections for refinement. *R*-factors based on *F*^2^ are statistically about twice as large as those based on *F*, and *R*- factors based on ALL data will be even larger.

### Fractional atomic coordinates and isotropic or equivalent isotropic displacement parameters (Å^2^)

**Table:**

	*x*	*y*	*z*	*U*_iso_*/*U*_eq_	
S1	0.42601 (4)	0.14356 (4)	0.65021 (3)	0.0548 (2)	
S2	0.43044 (5)	0.28236 (4)	0.71302 (4)	0.0658 (3)	
S3	0.91769 (4)	0.01716 (4)	0.58927 (3)	0.0549 (2)	
S4	0.95474 (4)	0.14921 (4)	0.52137 (3)	0.0595 (2)	
O1	0.50929 (13)	0.14107 (13)	0.66501 (12)	0.0812 (7)	
O2	0.40126 (15)	0.11824 (12)	0.59213 (9)	0.0745 (6)	
O3	0.39709 (17)	0.25589 (12)	0.76788 (9)	0.0842 (7)	
O4	0.51574 (14)	0.28768 (13)	0.70805 (14)	0.0964 (8)	
O5	0.99638 (12)	−0.01180 (13)	0.58338 (9)	0.0716 (6)	
O6	0.90029 (12)	0.05709 (11)	0.64260 (8)	0.0626 (5)	
O7	1.03467 (12)	0.13461 (14)	0.54114 (11)	0.0783 (7)	
O8	0.94424 (13)	0.17500 (12)	0.46090 (9)	0.0680 (6)	
C1	0.2418 (2)	0.0672 (2)	0.65607 (16)	0.0807 (10)	
H1	0.2706	0.0376	0.6309	0.097*	
C2	0.1600 (3)	0.0728 (3)	0.6496 (2)	0.1075 (15)	
H2	0.1340	0.0469	0.6200	0.129*	
C3	0.1172 (3)	0.1152 (3)	0.6855 (3)	0.1045 (15)	
H3	0.0619	0.1186	0.6805	0.125*	
C4	0.1543 (2)	0.1535 (2)	0.7294 (2)	0.0925 (12)	
H4	0.1244	0.1825	0.7544	0.111*	
C5	0.2375 (2)	0.14881 (17)	0.73661 (16)	0.0702 (9)	
H5	0.2631	0.1750	0.7662	0.084*	
C6	0.28160 (17)	0.10547 (14)	0.69983 (12)	0.0520 (7)	
C7	0.37104 (17)	0.09921 (14)	0.70854 (12)	0.0516 (7)	
H7	0.3846	0.1210	0.7473	0.062*	
C8	0.4035 (2)	0.02521 (17)	0.70958 (16)	0.0789 (10)	
H8A	0.3753	−0.0013	0.7396	0.118*	
H8B	0.4597	0.0261	0.7192	0.118*	
H8C	0.3960	0.0042	0.6706	0.118*	
C9	0.39327 (17)	0.23133 (14)	0.65224 (12)	0.0521 (7)	
H9A	0.4088	0.2534	0.6145	0.063*	
H9B	0.3351	0.2316	0.6541	0.063*	
C10	0.3892 (2)	0.36687 (17)	0.69637 (16)	0.0721 (9)	
H10	0.4176	0.3848	0.6608	0.086*	
C11	0.4098 (3)	0.4138 (2)	0.7492 (2)	0.1226 (18)	
H11A	0.3917	0.4601	0.7407	0.184*	
H11B	0.4669	0.4141	0.7551	0.184*	
H11C	0.3840	0.3970	0.7851	0.184*	
C12	0.30164 (19)	0.36350 (15)	0.68054 (15)	0.0607 (8)	
C13	0.2783 (2)	0.3767 (2)	0.62202 (18)	0.0861 (11)	
H13	0.3168	0.3885	0.5933	0.103*	
C14	0.1986 (4)	0.3726 (3)	0.6053 (3)	0.133 (2)	
H14	0.1825	0.3822	0.5659	0.160*	
C15	0.1430 (3)	0.3535 (3)	0.6495 (5)	0.149 (3)	
H15	0.0892	0.3485	0.6393	0.179*	
C16	0.1663 (4)	0.3425 (3)	0.7057 (4)	0.133 (2)	
H16	0.1279	0.3318	0.7348	0.160*	
C17	0.2446 (3)	0.34631 (19)	0.7224 (2)	0.0892 (12)	
H17	0.2594	0.3372	0.7622	0.107*	
C18	0.7175 (2)	−0.03355 (18)	0.52485 (15)	0.0732 (9)	
H18	0.7416	−0.0530	0.4909	0.088*	
C19	0.6381 (2)	−0.0121 (2)	0.52199 (19)	0.0934 (12)	
H19	0.6089	−0.0180	0.4865	0.112*	
C20	0.6028 (2)	0.0175 (2)	0.5712 (2)	0.0885 (11)	
H20	0.5498	0.0324	0.5691	0.106*	
C21	0.6446 (2)	0.02539 (19)	0.62323 (18)	0.0764 (10)	
H21	0.6202	0.0459	0.6566	0.092*	
C22	0.72306 (19)	0.00328 (17)	0.62717 (14)	0.0654 (8)	
H22	0.7509	0.0081	0.6634	0.078*	
C23	0.76090 (17)	−0.02629 (15)	0.57724 (13)	0.0536 (7)	
C24	0.84634 (17)	−0.05169 (15)	0.58011 (13)	0.0570 (7)	
H24	0.8581	−0.0748	0.5416	0.068*	
C25	0.8621 (2)	−0.10406 (19)	0.63070 (16)	0.0807 (10)	
H25A	0.8535	−0.0823	0.6692	0.121*	
H25B	0.9166	−0.1201	0.6282	0.121*	
H25C	0.8262	−0.1427	0.6264	0.121*	
C26	0.89830 (16)	0.07083 (16)	0.52489 (11)	0.0534 (7)	
H26A	0.9092	0.0441	0.4886	0.064*	
H26B	0.8417	0.0825	0.5246	0.064*	
C27	0.90765 (19)	0.20848 (18)	0.57385 (14)	0.0671 (9)	
H27	0.9089	0.1869	0.6140	0.081*	
C28	0.9598 (3)	0.2734 (3)	0.5763 (2)	0.1152 (16)	
H28A	0.9649	0.2925	0.5364	0.173*	
H28B	1.0120	0.2615	0.5916	0.173*	
H28C	0.9354	0.3070	0.6026	0.173*	
C29	0.82128 (19)	0.22151 (16)	0.55802 (14)	0.0611 (8)	
C30	0.7612 (2)	0.18929 (19)	0.59077 (16)	0.0771 (10)	
H30	0.7748	0.1587	0.6217	0.093*	
C31	0.6814 (2)	0.2018 (2)	0.5783 (2)	0.0978 (13)	
H31	0.6419	0.1802	0.6013	0.117*	
C32	0.6603 (3)	0.2448 (2)	0.5333 (2)	0.1013 (14)	
H32	0.6063	0.2530	0.5256	0.122*	
C33	0.7169 (3)	0.2765 (2)	0.4991 (2)	0.0950 (13)	
H33	0.7016	0.3052	0.4674	0.114*	
C34	0.7977 (3)	0.26611 (18)	0.51137 (16)	0.0802 (10)	
H34	0.8364	0.2889	0.4884	0.096*	

### Atomic displacement parameters (Å^2^)

**Table:**

	*U*^11^	*U*^22^	*U*^33^	*U*^12^	*U*^13^	*U*^23^
S1	0.0510 (4)	0.0588 (5)	0.0545 (4)	0.0056 (3)	0.0035 (3)	−0.0007 (3)
S2	0.0669 (5)	0.0562 (5)	0.0742 (6)	−0.0133 (4)	−0.0189 (4)	0.0005 (4)
S3	0.0473 (4)	0.0713 (5)	0.0460 (4)	0.0054 (3)	−0.0008 (3)	0.0045 (3)
S4	0.0458 (4)	0.0806 (6)	0.0522 (4)	−0.0071 (4)	0.0008 (3)	0.0088 (4)
O1	0.0452 (12)	0.0881 (17)	0.1104 (18)	0.0122 (11)	0.0044 (12)	−0.0007 (14)
O2	0.1017 (18)	0.0751 (15)	0.0467 (12)	0.0035 (13)	0.0062 (11)	−0.0084 (10)
O3	0.125 (2)	0.0704 (15)	0.0570 (13)	−0.0155 (14)	−0.0186 (13)	−0.0040 (11)
O4	0.0565 (14)	0.0835 (17)	0.149 (2)	−0.0166 (13)	−0.0312 (14)	0.0037 (16)
O5	0.0459 (12)	0.0994 (17)	0.0696 (13)	0.0169 (11)	−0.0043 (10)	0.0080 (12)
O6	0.0709 (14)	0.0757 (14)	0.0413 (10)	−0.0014 (11)	0.0021 (9)	−0.0015 (9)
O7	0.0431 (12)	0.1102 (19)	0.0816 (15)	−0.0108 (12)	−0.0066 (10)	0.0171 (13)
O8	0.0707 (14)	0.0814 (15)	0.0518 (12)	0.0013 (11)	0.0071 (10)	0.0163 (10)
C1	0.071 (2)	0.091 (3)	0.081 (2)	−0.0129 (19)	−0.0115 (18)	−0.009 (2)
C2	0.073 (3)	0.129 (4)	0.120 (4)	−0.028 (3)	−0.027 (3)	0.003 (3)
C3	0.058 (2)	0.115 (4)	0.140 (4)	−0.012 (3)	−0.007 (3)	0.032 (3)
C4	0.069 (2)	0.090 (3)	0.119 (3)	0.001 (2)	0.033 (2)	0.012 (2)
C5	0.061 (2)	0.069 (2)	0.080 (2)	−0.0048 (16)	0.0154 (17)	−0.0030 (17)
C6	0.0534 (16)	0.0508 (16)	0.0517 (16)	−0.0087 (13)	0.0000 (13)	0.0073 (12)
C7	0.0582 (17)	0.0513 (16)	0.0453 (15)	0.0014 (13)	−0.0045 (12)	0.0034 (12)
C8	0.101 (3)	0.0556 (19)	0.080 (2)	0.0167 (18)	−0.0067 (19)	0.0087 (16)
C9	0.0525 (16)	0.0547 (16)	0.0492 (15)	−0.0053 (13)	−0.0019 (12)	0.0091 (12)
C10	0.073 (2)	0.0572 (19)	0.086 (2)	−0.0122 (17)	−0.0067 (18)	0.0010 (16)
C11	0.143 (4)	0.065 (2)	0.160 (4)	−0.013 (3)	−0.052 (3)	−0.033 (3)
C12	0.0620 (19)	0.0499 (16)	0.070 (2)	−0.0009 (14)	0.0113 (15)	−0.0139 (14)
C13	0.087 (3)	0.092 (3)	0.079 (3)	0.019 (2)	0.000 (2)	−0.015 (2)
C14	0.119 (4)	0.134 (4)	0.147 (5)	0.058 (4)	−0.058 (4)	−0.071 (4)
C15	0.065 (3)	0.116 (4)	0.267 (9)	0.003 (3)	−0.021 (5)	−0.110 (6)
C16	0.085 (4)	0.085 (3)	0.229 (7)	−0.012 (3)	0.061 (4)	−0.036 (4)
C17	0.086 (3)	0.073 (2)	0.108 (3)	0.008 (2)	0.037 (2)	−0.010 (2)
C18	0.065 (2)	0.084 (2)	0.070 (2)	0.0042 (18)	−0.0046 (16)	−0.0184 (17)
C19	0.069 (2)	0.111 (3)	0.100 (3)	0.003 (2)	−0.023 (2)	−0.027 (2)
C20	0.053 (2)	0.092 (3)	0.121 (3)	0.0045 (19)	−0.004 (2)	−0.018 (2)
C21	0.061 (2)	0.078 (2)	0.090 (3)	0.0038 (18)	0.0190 (19)	−0.0103 (19)
C22	0.066 (2)	0.070 (2)	0.0601 (18)	0.0020 (16)	0.0102 (15)	−0.0033 (15)
C23	0.0514 (16)	0.0561 (17)	0.0535 (16)	−0.0020 (13)	0.0050 (13)	−0.0014 (13)
C24	0.0564 (18)	0.0613 (18)	0.0534 (16)	0.0067 (14)	0.0012 (13)	−0.0014 (13)
C25	0.090 (3)	0.068 (2)	0.084 (2)	0.0091 (19)	−0.0015 (19)	0.0184 (18)
C26	0.0425 (15)	0.0736 (19)	0.0442 (15)	0.0020 (14)	−0.0010 (11)	0.0033 (13)
C27	0.068 (2)	0.082 (2)	0.0521 (17)	−0.0144 (17)	0.0001 (15)	−0.0049 (15)
C28	0.099 (3)	0.120 (4)	0.126 (4)	−0.050 (3)	0.006 (3)	−0.042 (3)
C29	0.0656 (19)	0.0579 (17)	0.0597 (18)	−0.0021 (15)	0.0042 (15)	−0.0043 (14)
C30	0.071 (2)	0.070 (2)	0.090 (2)	0.0053 (18)	0.0155 (18)	0.0123 (18)
C31	0.070 (3)	0.077 (3)	0.146 (4)	0.005 (2)	0.023 (2)	0.003 (3)
C32	0.081 (3)	0.085 (3)	0.138 (4)	0.025 (2)	−0.011 (3)	−0.019 (3)
C33	0.117 (4)	0.078 (3)	0.091 (3)	0.041 (3)	−0.015 (3)	−0.002 (2)
C34	0.106 (3)	0.065 (2)	0.069 (2)	0.008 (2)	0.014 (2)	0.0018 (17)

### Geometric parameters (Å, °)

**Table:**

S1—O1	1.426 (2)	C13—H13	0.9300
S1—O2	1.433 (2)	C14—C15	1.396 (9)
S1—C9	1.778 (3)	C14—H14	0.9300
S1—C7	1.797 (3)	C15—C16	1.318 (9)
S2—O3	1.428 (2)	C15—H15	0.9300
S2—O4	1.429 (2)	C16—C17	1.358 (7)
S2—C9	1.776 (3)	C16—H16	0.9300
S2—C10	1.805 (3)	C17—H17	0.9300
S3—O5	1.431 (2)	C18—C23	1.372 (4)
S3—O6	1.437 (2)	C18—C19	1.388 (5)
S3—C26	1.788 (3)	C18—H18	0.9300
S3—C24	1.793 (3)	C19—C20	1.362 (5)
S4—O7	1.430 (2)	C19—H19	0.9300
S4—O8	1.436 (2)	C20—C21	1.352 (5)
S4—C26	1.781 (3)	C20—H20	0.9300
S4—C27	1.807 (3)	C21—C22	1.377 (4)
C1—C2	1.374 (5)	C21—H21	0.9300
C1—C6	1.385 (4)	C22—C23	1.392 (4)
C1—H1	0.9300	C22—H22	0.9300
C2—C3	1.345 (6)	C23—C24	1.507 (4)
C2—H2	0.9300	C24—C25	1.529 (4)
C3—C4	1.366 (6)	C24—H24	0.9800
C3—H3	0.9300	C25—H25A	0.9600
C4—C5	1.398 (5)	C25—H25B	0.9600
C4—H4	0.9300	C25—H25C	0.9600
C5—C6	1.377 (4)	C26—H26A	0.9700
C5—H5	0.9300	C26—H26B	0.9700
C6—C7	1.507 (4)	C27—C29	1.502 (4)
C7—C8	1.526 (4)	C27—C28	1.524 (5)
C7—H7	0.9800	C27—H27	0.9800
C8—H8A	0.9600	C28—H28A	0.9600
C8—H8B	0.9600	C28—H28B	0.9600
C8—H8C	0.9600	C28—H28C	0.9600
C9—H9A	0.9700	C29—C30	1.383 (4)
C9—H9B	0.9700	C29—C34	1.398 (5)
C10—C12	1.501 (4)	C30—C31	1.379 (5)
C10—C11	1.517 (5)	C30—H30	0.9300
C10—H10	0.9800	C31—C32	1.341 (6)
C11—H11A	0.9600	C31—H31	0.9300
C11—H11B	0.9600	C32—C33	1.353 (6)
C11—H11C	0.9600	C32—H32	0.9300
C12—C17	1.367 (5)	C33—C34	1.388 (5)
C12—C13	1.374 (5)	C33—H33	0.9300
C13—C14	1.382 (6)	C34—H34	0.9300
			
O1—S1—O2	118.26 (15)	C13—C14—C15	117.8 (6)
O1—S1—C9	108.95 (14)	C13—C14—H14	121.1
O2—S1—C9	104.96 (13)	C15—C14—H14	121.1
O1—S1—C7	108.39 (14)	C16—C15—C14	120.5 (6)
O2—S1—C7	109.48 (14)	C16—C15—H15	119.8
C9—S1—C7	106.14 (13)	C14—C15—H15	119.8
O3—S2—O4	118.55 (16)	C15—C16—C17	122.0 (6)
O3—S2—C9	107.97 (13)	C15—C16—H16	119.0
O4—S2—C9	109.17 (16)	C17—C16—H16	119.0
O3—S2—C10	110.31 (17)	C16—C17—C12	119.9 (5)
O4—S2—C10	107.35 (16)	C16—C17—H17	120.1
C9—S2—C10	102.27 (14)	C12—C17—H17	120.1
O5—S3—O6	117.93 (13)	C23—C18—C19	120.7 (3)
O5—S3—C26	108.59 (13)	C23—C18—H18	119.6
O6—S3—C26	107.82 (13)	C19—C18—H18	119.6
O5—S3—C24	107.99 (14)	C20—C19—C18	120.0 (3)
O6—S3—C24	110.80 (13)	C20—C19—H19	120.0
C26—S3—C24	102.61 (13)	C18—C19—H19	120.0
O7—S4—O8	117.77 (13)	C21—C20—C19	120.2 (3)
O7—S4—C26	108.16 (14)	C21—C20—H20	119.9
O8—S4—C26	105.66 (13)	C19—C20—H20	119.9
O7—S4—C27	109.44 (15)	C20—C21—C22	120.5 (3)
O8—S4—C27	108.98 (15)	C20—C21—H21	119.7
C26—S4—C27	106.18 (14)	C22—C21—H21	119.7
C2—C1—C6	120.4 (4)	C21—C22—C23	120.4 (3)
C2—C1—H1	119.8	C21—C22—H22	119.8
C6—C1—H1	119.8	C23—C22—H22	119.8
C3—C2—C1	120.8 (4)	C18—C23—C22	118.1 (3)
C3—C2—H2	119.6	C18—C23—C24	120.0 (3)
C1—C2—H2	119.6	C22—C23—C24	121.8 (3)
C2—C3—C4	120.4 (4)	C23—C24—C25	114.1 (3)
C2—C3—H3	119.8	C23—C24—S3	113.0 (2)
C4—C3—H3	119.8	C25—C24—S3	107.0 (2)
C3—C4—C5	119.7 (4)	C23—C24—H24	107.5
C3—C4—H4	120.2	C25—C24—H24	107.5
C5—C4—H4	120.2	S3—C24—H24	107.5
C6—C5—C4	120.0 (3)	C24—C25—H25A	109.5
C6—C5—H5	120.0	C24—C25—H25B	109.5
C4—C5—H5	120.0	H25A—C25—H25B	109.5
C5—C6—C1	118.6 (3)	C24—C25—H25C	109.5
C5—C6—C7	120.0 (3)	H25A—C25—H25C	109.5
C1—C6—C7	121.3 (3)	H25B—C25—H25C	109.5
C6—C7—C8	115.3 (3)	S4—C26—S3	115.46 (15)
C6—C7—S1	111.98 (18)	S4—C26—H26A	108.4
C8—C7—S1	105.9 (2)	S3—C26—H26A	108.4
C6—C7—H7	107.8	S4—C26—H26B	108.4
C8—C7—H7	107.8	S3—C26—H26B	108.4
S1—C7—H7	107.8	H26A—C26—H26B	107.5
C7—C8—H8A	109.5	C29—C27—C28	114.7 (3)
C7—C8—H8B	109.5	C29—C27—S4	111.9 (2)
H8A—C8—H8B	109.5	C28—C27—S4	107.1 (2)
C7—C8—H8C	109.5	C29—C27—H27	107.6
H8A—C8—H8C	109.5	C28—C27—H27	107.6
H8B—C8—H8C	109.5	S4—C27—H27	107.6
S2—C9—S1	116.03 (15)	C27—C28—H28A	109.5
S2—C9—H9A	108.3	C27—C28—H28B	109.5
S1—C9—H9A	108.3	H28A—C28—H28B	109.5
S2—C9—H9B	108.3	C27—C28—H28C	109.5
S1—C9—H9B	108.3	H28A—C28—H28C	109.5
H9A—C9—H9B	107.4	H28B—C28—H28C	109.5
C12—C10—C11	115.2 (3)	C30—C29—C34	117.3 (3)
C12—C10—S2	112.2 (2)	C30—C29—C27	119.8 (3)
C11—C10—S2	107.2 (3)	C34—C29—C27	122.9 (3)
C12—C10—H10	107.3	C31—C30—C29	121.0 (4)
C11—C10—H10	107.3	C31—C30—H30	119.5
S2—C10—H10	107.3	C29—C30—H30	119.5
C10—C11—H11A	109.5	C32—C31—C30	120.6 (4)
C10—C11—H11B	109.5	C32—C31—H31	119.7
H11A—C11—H11B	109.5	C30—C31—H31	119.7
C10—C11—H11C	109.5	C31—C32—C33	120.6 (4)
H11A—C11—H11C	109.5	C31—C32—H32	119.7
H11B—C11—H11C	109.5	C33—C32—H32	119.7
C17—C12—C13	119.0 (4)	C32—C33—C34	120.2 (4)
C17—C12—C10	121.9 (3)	C32—C33—H33	119.9
C13—C12—C10	119.1 (3)	C34—C33—H33	119.9
C12—C13—C14	120.9 (4)	C33—C34—C29	120.3 (4)
C12—C13—H13	119.6	C33—C34—H34	119.9
C14—C13—H13	119.6	C29—C34—H34	119.9
			
C6—C1—C2—C3	−0.1 (7)	C23—C18—C19—C20	1.1 (6)
C1—C2—C3—C4	−0.3 (7)	C18—C19—C20—C21	−0.9 (7)
C2—C3—C4—C5	0.6 (7)	C19—C20—C21—C22	−0.3 (6)
C3—C4—C5—C6	−0.5 (6)	C20—C21—C22—C23	1.2 (5)
C4—C5—C6—C1	0.1 (5)	C19—C18—C23—C22	−0.2 (5)
C4—C5—C6—C7	−178.6 (3)	C19—C18—C23—C24	178.3 (3)
C2—C1—C6—C5	0.2 (5)	C21—C22—C23—C18	−1.0 (5)
C2—C1—C6—C7	178.9 (3)	C21—C22—C23—C24	−179.4 (3)
C5—C6—C7—C8	132.9 (3)	C18—C23—C24—C25	−123.5 (3)
C1—C6—C7—C8	−45.7 (4)	C22—C23—C24—C25	54.9 (4)
C5—C6—C7—S1	−105.8 (3)	C18—C23—C24—S3	114.0 (3)
C1—C6—C7—S1	75.5 (3)	C22—C23—C24—S3	−67.6 (3)
O1—S1—C7—C6	174.3 (2)	O5—S3—C24—C23	−172.48 (19)
O2—S1—C7—C6	−55.4 (2)	O6—S3—C24—C23	57.0 (2)
C9—S1—C7—C6	57.4 (2)	C26—S3—C24—C23	−57.9 (2)
O1—S1—C7—C8	−59.3 (2)	O5—S3—C24—C25	61.2 (2)
O2—S1—C7—C8	71.1 (2)	O6—S3—C24—C25	−69.4 (2)
C9—S1—C7—C8	−176.2 (2)	C26—S3—C24—C25	175.8 (2)
O3—S2—C9—S1	−67.0 (2)	O7—S4—C26—S3	39.8 (2)
O4—S2—C9—S1	63.2 (2)	O8—S4—C26—S3	166.74 (15)
C10—S2—C9—S1	176.68 (17)	C27—S4—C26—S3	−77.6 (2)
O1—S1—C9—S2	−41.5 (2)	O5—S3—C26—S4	−69.9 (2)
O2—S1—C9—S2	−169.10 (16)	O6—S3—C26—S4	58.95 (19)
C7—S1—C9—S2	75.01 (19)	C24—S3—C26—S4	175.96 (16)
O3—S2—C10—C12	−67.9 (3)	O7—S4—C27—C29	−175.6 (2)
O4—S2—C10—C12	161.6 (2)	O8—S4—C27—C29	54.3 (3)
C9—S2—C10—C12	46.8 (3)	C26—S4—C27—C29	−59.1 (3)
O3—S2—C10—C11	59.5 (3)	O7—S4—C27—C28	57.9 (3)
O4—S2—C10—C11	−71.0 (3)	O8—S4—C27—C28	−72.2 (3)
C9—S2—C10—C11	174.2 (3)	C26—S4—C27—C28	174.4 (3)
C11—C10—C12—C17	−54.8 (4)	C28—C27—C29—C30	−134.8 (3)
S2—C10—C12—C17	68.2 (4)	S4—C27—C29—C30	103.0 (3)
C11—C10—C12—C13	126.6 (4)	C28—C27—C29—C34	44.4 (4)
S2—C10—C12—C13	−110.4 (3)	S4—C27—C29—C34	−77.8 (4)
C17—C12—C13—C14	−0.2 (5)	C34—C29—C30—C31	−1.0 (5)
C10—C12—C13—C14	178.5 (3)	C27—C29—C30—C31	178.2 (3)
C12—C13—C14—C15	−1.1 (7)	C29—C30—C31—C32	1.0 (6)
C13—C14—C15—C16	2.5 (8)	C30—C31—C32—C33	0.4 (7)
C14—C15—C16—C17	−2.8 (9)	C31—C32—C33—C34	−1.8 (6)
C15—C16—C17—C12	1.5 (7)	C32—C33—C34—C29	1.7 (6)
C13—C12—C17—C16	0.0 (5)	C30—C29—C34—C33	−0.3 (5)
C10—C12—C17—C16	−178.6 (4)	C27—C29—C34—C33	−179.5 (3)
